# Removal of heavy metal iron(II) ions from wastewater using an ultrasonic system with climbazole-alcohol

**DOI:** 10.55730/1300-0527.3729

**Published:** 2025-03-12

**Authors:** Melek GÖKMEN KARAKAYA, Bahdişen GEZER, Abdullah MENZEK, Özlem GÜNDOĞDU AYTAÇ

**Affiliations:** 1 Laboratory Technology Program, Department of Chemistry and Chemical Processing Technologies, Banaz Vocational School, Uşak University, Uşak, Turkiye; 2 Electrical Electronics Program, Department of Electrical Electronics Engineering, Faculty of Engineering and Natural Sciences, Uşak University, Uşak, Turkiye; 3 Emergency Aid and Disaster Management Program, Department of Emergency Aid and Disaster Management, Faculty of Health Sciences, Ardahan University, Ardahan, Turkiye; 4 Food Technology Program, Department of Food Processing, Kaman Vocational School, Kırşehir Ahi Evran University, Kırşehir, Turkiye

**Keywords:** Climbazole-alcohol, sodium borohydride, response surface methodology, optimization, iron(ll) ions, wastewater treatment

## Abstract

Climbazole (CBZ) is an antifungal active pharmaceutical ingredient often used in antidandruff products. In this study, the ketone group in the racemic CBZ molecule was reduced to synthesize CBZ-alcohol, named 1-(4-chlorophenoxy)-1-(1*H*-imidazol-1-yl)-3,3-dimethylbutan-2-ol, and the optimum adsorption conditions were investigated for removing iron(II) (Fe^2+^) ions from wastewater by adsorption from aqueous solutions using the economical and environmentally friendly ultrasonic method. The parameters and levels used in the study were designed using response surface methodology and model equations were derived to optimize the results. The independent variables selected were the initial pH (1, 3, and 5), adsorption time (30, 45, and 60 min), adsorption temperature (40, 60, and 80 °C), and Fe^2+^ ions consumption percentage from the wastewater. Experiments were conducted on a real wastewater sample taken from the Uşak Organized Industrial Zone. Conditions that maximize each dependent variable were specified separately and verification experiments were conducted under these conditions. Maximum Fe^2+^ ion consumption was 91.83%. The R^2^ value of the model was 0.9598. The findings demonstrate that CBZ-alcohol was effective as an adsorbent in Fe^2+^ ion removal from aqueous solution.

## 1. Introduction

Climbazole (CBZ) is widely used in pharmaceutical and personal care products [1–3]. Various studies have been conducted on the separation of CBZ from wastewater [4–6]. The removal rate of CBZ mixed into wastewater is generally low in wastewater treatment plants [5,6]. The freshwater microalga *Scenedesmus obliquus* converted CBZ into CBZ-alcohol via biotransformation and the toxicity of the latter was much lower than that of the former [7]. In synthetic organic chemistry, there are similar reduction reactions with sodium borohydride in water [8].

In the present study, diastereomeric 1-(4-chlorophenoxy)-1-(1-imidazolyl)-3,3-dimethyl-2-butanols (CBZ-alcohol) were synthesized by reduction reaction of the ketone group in CBZ. The metal retention capacity of the obtained mixture of CBZ-alcohol in industrial wastewater was examined. The waste generated by industry causes water pollution. Industrial pollution disrupts the structure of soil, hinders the development of plants, causes mutations in living organisms, and increases the risk of diseases such as cancer [9]. Heavy metals have a density greater than 5 g/cm^3^ [10]. Lead, mercury, cadmium, arsenic, and chromium are some common heavy metals used in different industries [11]. The release of heavy metal ions in water and the environment above the limits set by the World Health Organization (WHO) affects flora and fauna, and also causes serious diseases such as liver and neurological disorders in humans [12].

Traditional treatment technologies commonly used for wastewater include chemical precipitation, membrane filtration, electrochemical treatment, and adsorption—the latter being the most efficient [13]. Adsorption is cost-effective, easy to use, and efficiently separates toxic organic and inorganic species from the aqueous environment. Furthermore, the disposal of the separated toxic material does not affect biological structures [14]. The most important criterion in adsorption technology is the desorption capacity of the adsorbent. An adsorbent with high desorption capacity means toxic and hazardous waste can be dealt with safely.

The ultrasonic process has a high radical production capacity in both low (20 kHz) and high frequency (>200 kHz) ranges. As a result of the explosion of cavitation bubbles created by ultrasonic waves in water, a large amount of energy at high temperature and pressure (5000 K, 1000 atm) is released. Organic compounds that are broken down by the effects of the cavitation bubbles can be transformed into harmless by-products and radicals are formed. Mass transfer from the electrode surface increases and the surface is cleaned as a result of the acoustic cavitation and shock waves generated by the ultrasonic effect. Ultrasonic shock wave cleansing of the electrode surface accelerates chemical reactivity and thus increases purification yield [15].

Response surface methodology (RSM) is a statistical and mathematical approach that combines various techniques to explore the relationships between response and independent variables. These variables can be studied individually or in combination to enhance, optimize, and develop processes [16]. RSM is used not only to examine the effects of independent variables but also to generate a mathematical model [17]. By employing RSM, it becomes possible to determine whether there are interactions between the parameters that influence the system, identify the dominant interactions (if any), and ascertain the sensitivity of the process to specific independent variables [18]. Equation 1 is a second-degree polynomial equation that showcases the variation in the response value [19].


(1) $$Y_{m}=b_{0}+\Sigma_{i=1}^{k}b_{i}X_{i}+\Sigma_{i=1}^{k}b_{ii}X_{i}^{2}+\Sigma_{i}^{i<j}\Sigma_{j}\;b_{ij}X_{i}X_{j}+ɛ$$

Y_m_ represents the response variable to be modeled; β_0_, β_i_, β_ii_, and β_ij_ represent the model coefficients; X_i_ and X_j_ represent the coded values of the independent variables; and ɛ represents the error term.

In accordance with Equation 1, the mathematical formulation of RSM encompasses linear parameters, quadratic relationships, and pairwise interactions. Over the past two decades, RSM has garnered significant attention as one of the prominent multivariate experimental design methods for designing, optimizing, and modeling experiments [20]. Optimization techniques have been used in numerous studies to effectively eliminate diverse pollutants using various processes including electrooxidation, the Fenton process, photo-Fenton process, and adsorption [21].

In the present study, optimization for Fe^2+^ ion adsorption from wastewater using an ultrasonic method with CBZ-alcohol was examined. Diastereomeric 1-(4-chlorophenoxy)-1-(1-imidazolyl)-3,3-dimethyl-2-butanols (CBZ-alcohol) was synthesized by a reduction reaction of the ketone group in CBZ. The metal retention capacity of CBZ-alcohol in industrial wastewater was examined. Unlike previous studies, our study was performed with ultrasound to provide more diffusion of CBZ-alcohol into the pores. The experiments were statistically designed and the effects of the parameters were examined. A statistical model was established using analysis of variance (ANOVA) between the surface area and the parameters, and the optimum process conditions were investigated by optimization using this model. These empirical models and optimum results can be used to determine the appropriate process conditions constituting a starting point for further studies on a larger scale and may also be an important source of information for feasibility studies.

## 2. Materials and methods

### 2.1. Chemical material and apparatus

All chemicals were purchased from Sigma-Aldrich and Merck. They were analytical grade and used without further purification steps. The reactions were visualized via thin-layer chromatography (TLC). ^1^H NMR and ^13^C NMR spectra were obtained at 400 MHz and 100 MHz, respectively, using CDCl_3_ and a Varian spectrometer (Palo Alto, USA).

The independent variables and their corresponding levels for the experimental study were established based on preliminary investigations and a review of the relevant literature. The experimental design was determined through RSM, and the experiments were conducted in accordance with the specified conditions outlined in the design.


(2) $$Heavy\;metal\;removal\;yield\;(\%)=\frac{C_{0}-C_{s}}{C_{0}}\times 100$$

In equation 2, C_0_ is the initial pollutant concentration (mg/L, Pt-Co) and C_S_ is the pollutant concentration remaining in the environment at the end of the experiment (mg/L, Pt-Co).

Wastewater for treatment was obtained from distribution and collection pipes. Samples were taken from the inlet and outlet manhole of each cell. Seventeen 100 mL samples of wastewater solutions were mixed at room temperature and a speed of 100 rpm. After 60 min, the speed was reduced to 50 rpm and the final pH value for neutralization was corrected to around 8 with sodium hydroxide (NaOH). The solutions were left to stand without stirring for 60 min to allow the precipitation process to take place. Subsequently, 25 mL of sample taken from the upper clear phase of the precipitated mixture was added to 100 mL glass beakers containing some manganese dioxide (MnO_2_) to eliminate the remaining hydrogen peroxide (H_2_O_2_). The wastewater was then filtered through a suspended solids apparatus and prepared for color measurement. Adsorption experiments with 17 wastewater solutions using the ultrasonic wave process were carried out under the conditions specified in Table 1.

**Figure 1:**
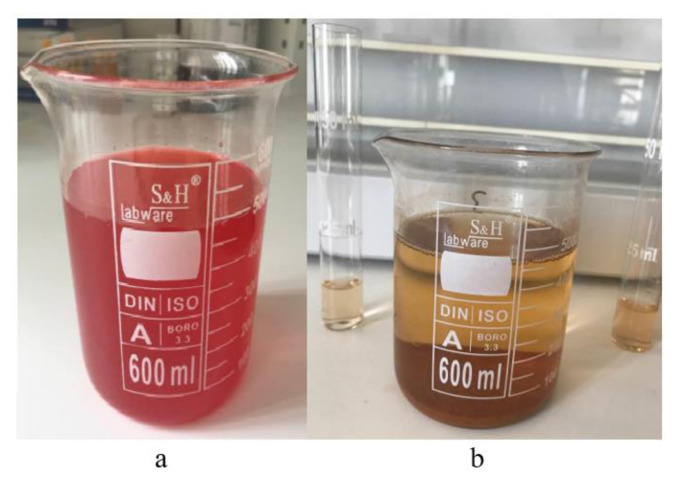
The real wastewater before (a) and after (b) the application of the adsorption method.

The difference in the color of the wastewater taken from Uşak Organized Industrial Zone before and after the application of the adsorption process can be seen very clearly in Figure 1.

The findings related to the textile wastewater are given in Table 1. Average concentrations of Fe^2+^ions, treatment efficiencies and standard deviations are given. There is a notable reduction in Fe^2+^ ions after adsorption with CBZ-alcohol (Table 1).

### 2.2. Synthesis of 1-(4-chlorophenoxy)-1-(1*H*-imidazol-1-yl)-3,3-dimethylbutan-2-ol

**Figure 2:**
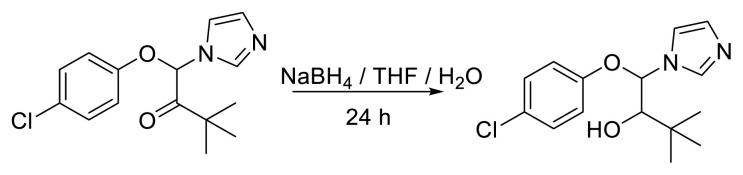
The equation showing the reduction reaction of CBZ to CBZ-alcohol with sodium borohydride.

CBZ (1000 mg, 3.416 mmol) and sodium borohydride (NaBH_4_) (387.655 mg, 10.247 mmol) were placed in a 100 mL round-bottom flask and dissolved in 20 mL tetrahydrofuran and 20 mL water. The resulting mixture was magnetically stirred at room temperature for 24 h. It was checked by TLC. The reaction mixture was extracted with ethyl acetate (2 × 30 mL). After the organic phases were combined and dried over sodium sulfate, they were filtered and the solvent was evaporated under reduced pressure. Subsequent purification of the crude residue by crystallization with ethyl acetate/hexane provided the desired products. CBZ-alcohol, named 1-(4-chlorophenoxy)-1-(1*H*-imidazol-1-yl)-3,3-dimethylbutan-2-ol or 1-(4-chlorophenoxy)-1-(1-imidazolyl)-3,3-dimethyl-2-butanol, was obtained. The reduction reaction equation is shown in Figure 2. CBZ-alcohol is known to be formed by the reduction of CBZ [7,22].

### 2.3. Experimental design

The independent variables used were initial pH (X_1_), time (X_2_), and temperature (X_3_) at three different levels each. The design levels and values where the outlying points are located are given in Table 2.

### 2.4. Adsorption mechanism

Different methods such as adsorption, biosorption, bioremediation, coagulation and flocculation, oxidation, precipitation, membrane technology, electrochemical processes, ion exchange, and photocatalysis are used to remove metals from wastewater [23,24]. Of these methods, adsorption is one of the most popular because it is inexpensive, easy to apply, and highly efficient [25]. The distribution of the substance in solution changes when adsorption occurs at the liquid surface due to surface tension and electrostatic forces. According to Gibbs, substances that decrease the surface tension have a higher concentration at the interface than in the liquid, while substances that increase the surface tension have a lower concentration [26].

### 2.5. Use of ultrasound in wastewater treatment

After the acoustic cavitation, the pollutants in the wastewater are broken down by the hydroxyl radicals produced. The energy needed for chemical and physical reactions comes from the inward collapse of the bubbles formed during cavitation [27]. After the desired conditions are achieved, the organic pollutants in the wastewater are broken down in two ways: oxidation by hydroxyl radicals and pyrolysis. These two steps do not necessarily occur together continuously. Pyrolysis is usually a precursor in high-concentration solutions, while OH radicals are a precursor in low-concentration solutions. It is also important to determine the optimum conditions (such as time, frequency, and power), especially in terms of economics and applicability, since ultrasound can be affected by many parameters [28].

### 2.6. Fe^2+^ ion removal with CBZ-alcohol

First, 300 mg of CBZ-alcohol and 15 mL of wastewater prepared at different pH values were added to a flask. The adsorption experiments were carried out in an ultrasonic bath under the conditions specified in Table 3. The absorbance of Fe^2+^ ion solutions prepared at certain concentrations was measured by spectrophotometer. The concentration values obtained were calculated using Equation 2.

## 3. Results and discussion

### 3.1. Chemistry

The compound 1-(4-chlorophenoxy)-1-(1*H*-imidazol-1-yl)-3,3-dimethylbutan-2-ol was obtained by reduction of CBZ. The reaction was monitored by TLC. The resulting compounds were purified by using column chromatography. The ^1^H NMR spectrum of the synthesized compound is given in supplementary material.

### 3.2. Physical properties and spectral data of the synthesized compound

A 97% yield of 1-(4-chlorophenoxy)-1-(1*H*-imidazol-1-yl)-3,3-dimethylbutan-2-ol was obtained as a light yellow solid. ^1^H NMR (400 MHz, CDCl_3_): δ 7.56+7.49 (2xs, 1H), 7.21-7.14 (m, 2H), 7.13+7.09 (2xs, 1H), 6.94+6.98 (2xs, 1H), 6.77-6.69 (m, 2H), 5.83+5.65 (2xd, *J* = 2.6, 6.4 Hz, 1H), 4.90+4.36 (2xbp, 1H), 3.71+3.51 (2xd, *J* = 6.4, 2.6 Hz, 1H), 1.02+1.01 (2xs, 9H); ^13^C NMR (100 MHz, CDCl_3_): δ 154.15, 154.03, 136.15, 135.88, 129.87 (2C), 129.78 (2C), 129.41, 128.91, 128.89, 128.47, 118.66, 118.12, 117.76 (2C), 117.15 (2C), 87.02, 85.59, 80.58, 78.76, 34.84, 34.63, 26.45 (3 CH_3_), 26.42 (3 CH_3_); LC-Q-TOF-MS (m/z) calcd for [C_15_H_19_N_2_O_2_Cl + H]^+^: 295.1207; found: 295.1201 [22].

### 3.3. Application of ultrasound for removal of Fe^2+^ ions from wastewater with CBZ-alcohol

The removal of the heavy metal Fe^2+^ ions from wastewater using 300 mg of CBZ-alcohol, under the conditions described in Table 3, using an ultrasonic device operating at a frequency of 100 kHz was examined. Table 3 shows the Fe^2+^ ion percentage removal efficiencies obtained for each set of conditions in the RSM design matrix.

**Figure 3:**
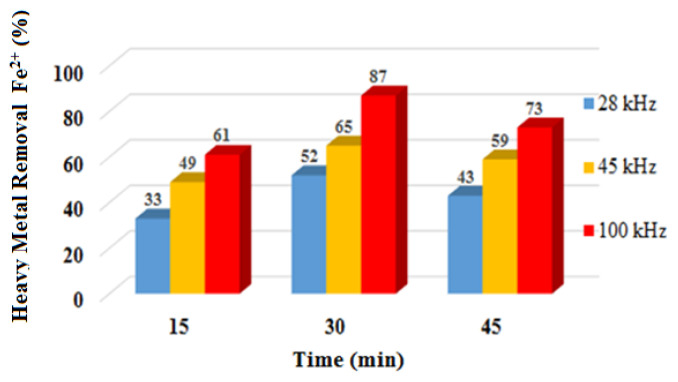
Effect of different ultrasonic frequencies on the removal of Fe^2+^ ions.

Wastewater containing Fe^2+^ ions was investigated at different time points in the ultrasound setup. For this purpose, the adsorption method was applied to wastewater in 100 mL amounts adjusted to 15, 30, and 45 min. The variation in absorbance values in the UV-vis spectrophotometer with time is given in Figure 3. Fe^2+^ ion removal efficiency increased significantly with ultrasound. Ultrasound at a frequency of 100 kHz resulted in significantly increased yields of nano-sized iron(II) oxide. This is because 100 kHz has been reported to be the optimal sound wave for Fe^2+^ ions. Fe^2+^ ion production causes a significant increase in extra hydroxyl radicals [29].

**Figure 4:**
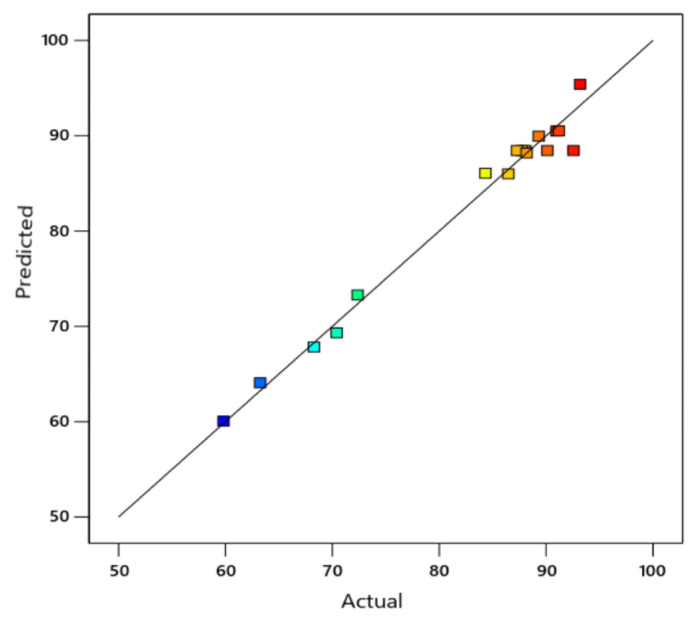
Correlation graphs of experimental and model data for Fe^2+^ ion removal efficiencies. (The distribution of the parameters used in the experiment is shown in different colors).

In Figure 4, a harmonious relationship can be seen between the experimental results and the model results. R^2^ and adjusted R^2^ values obtained from separately optimized models under maximum removal conditions are shown in Table 4. The predicted R^2^ of 0.6356 is not as close to the adjusted R^2^ of 0.9598 as one might normally expect, i.e. the difference is more than 0.2. This may indicate a large block effect or a possible problem with the model and/or data used. Factors to consider are model reduction, response transformation, outliers, etc. All empirical models should be tested by doing confirmation runs. Adeq precision was 20.910, indicating an adequate signal-to-noise ratio. The F-value of 43.49 implies the model is significant. There is only a 0.01% chance that an F-value this large could occur due to noise.

p-values less than 0.05 indicate that model terms are significant. In this case, X_1_, X_2_, X_3_, X_1_X_2_, X_1_^2^, X_2_^2^, and X_3_^2^ are significant model terms. Values greater than 0.1 indicate model terms are not significant. If there are too many insignificant model terms (not counting those required to support hierarchy), model reduction may improve the model used (Table 5) [30]. The lack-of-fit F-value of 1.02 implies the lack-of-fit is not significant relative to the error. There is a 47.06% chance that a lack-of-fit F-value this large could occur due to noise.

**Figure 5:**
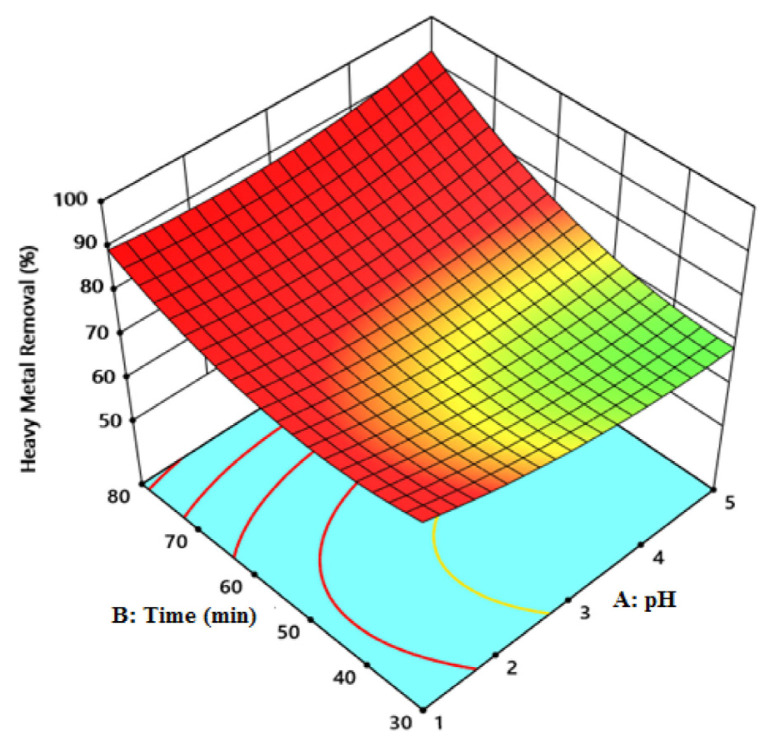
The effect of time and pH on Fe^2+^ ion adsorption capacity.

In adsorption, time and pH were important parameters (Figure 5). The pH of the environment affects the adsorption mechanism over time, affecting the binding points of the adsorbent with the pollutant. This influences the degree of ionization, surface charge, and the types of species adsorbed. Additionally, the degree of adsorption varies due to the variation in the concentration of dissolved species as a result of hydrolysis and the precipitation of Fe^2+^ ions depending on the pH value. The acidity of the environment greatly affects the binding of metal ions to active sites on the adsorbent surface over time due to the interaction between metal (Fe^2+^) and hydrogen ions [31].

**Figure 6:**
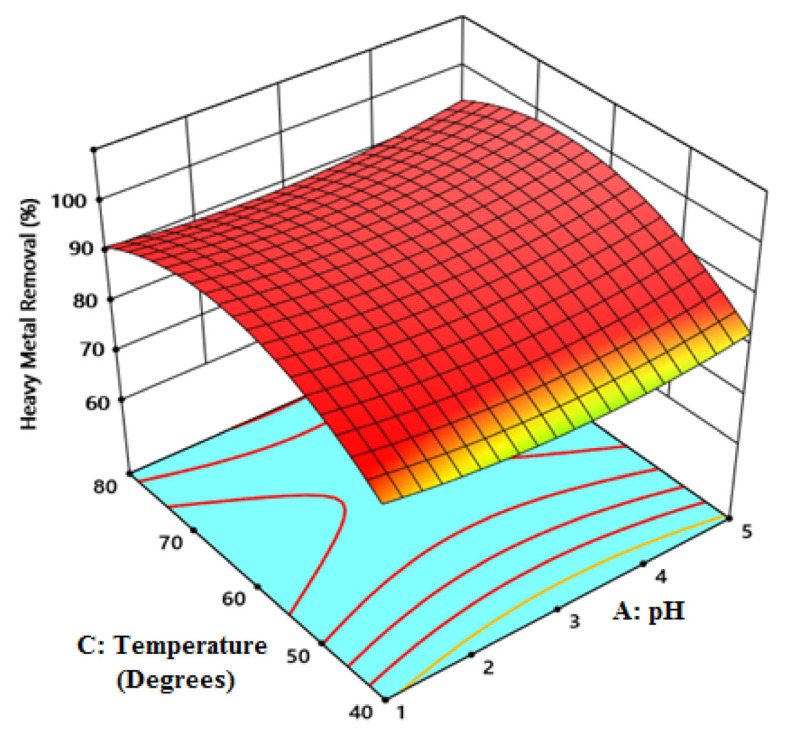
The effect of temperature and pH on Fe^2+^ ion adsorption capacity.

Figure 6 is a three-dimensional graph showing the effect of pH and temperature on Fe^2+^ ion adsorption capacity. Adsorption capacity decreases as temperature rises. The kinetic energy of Fe^2+^ ion particles increases with the temperature of the solution. This may have had a reverse effect on adsorption. At the same time, the decrease in the amount adsorbed may have damaged the active binding sites on the adsorbent [32]. This behavior indicates that the adsorption process is exothermic. Additionally, in the literature [33], it was stated that as the temperature increases, the surface of the adsorbent will become damaged, leading to a decrease in adsorption capacity.

**Figure 7:**
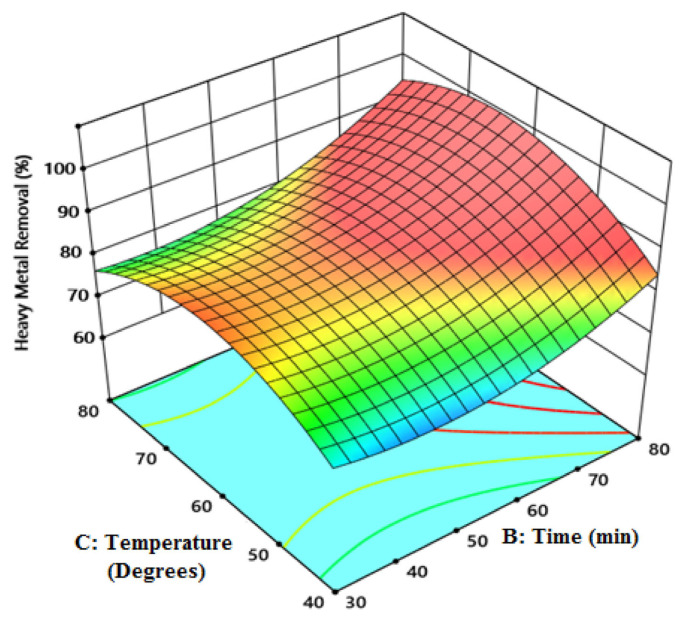
The effect of temperature and time on Fe^2+^ ion adsorption capacity.

Figure 7 shows that the adsorption capacity increases rapidly with an increase in Fe^2+^ ion concentration. At higher levels, the active sites on the surface are saturated with Fe^2+^ ions and adsorption is at equilibrium. This is the phase of gradual adsorption. As the Fe^2+^ ion concentration increases, the rate of increase in capacity also increases slowly, and then the metal ion uptake reaches equilibrium. Due to the increase in Fe^2+^ ions in the solution that cannot be adsorbed, the pores on the adsorbent surface become insufficient for the uptake of more Fe^2+^ ions. The observed decrease in Fe^2+^ ion adsorption is due to the increased electrostatic interactions with Fe^2+^ ions that are less attracted to the increasing metal ions. A certain period of time is required to achieve equilibrium in the adsorption process. When the active surface of the adsorbent is covered, further adsorption does not occur and, as a result, the adsorption capacity remains constant over time [34].

The model equation obtained for Fe^2+^ ion removal efficiency (%) is given below:


(3) $$\%\;{\text{Fe}}^{2+}\;\text{Removal}=+1.71241-21.61573{\text{X}}_{1}-2.52017{\text{X}}_{2}+5.71294{\text{X}}_{3}+0.146702{\text{X}}_{1}{\text{X}}_{2}+0.037598{\text{X}}_{1}{\text{X}}_{3}+0.007420{\text{X}}_{2}{\text{X}}_{3}+1.44629{{\text{X}}_{1}}^{2}+0.019984{{\text{X}}_{2}}^{2}-0.049409{{\text{X}}_{3}}^{2}$$

The equation can be used to make predictions about the response for given levels of each factor. Here the levels are specified in the original units for each factor. This equation should not be used to determine the relative impact of each factor because the coefficients are scaled to accommodate the units of each factor and the intercept is not at the center of the design space (Eq. 3).

The difference between RSM and existing optimization methods is the continuous renewal of the optimum result for each uncontrollable variable that changes instantly [35]. In addition, by using the obtained mathematical models, it is possible to calculate the change in output under different conditions in the determined level range of dependent variables without conducting experiments.

The optimum conditions determined for maximum removal efficiency of water containing heavy metals were: pH 2.95, 73.91 min, and 72.95 °C (Table 6). Outside the determined optimum, the removal efficiency of Fe^2+^ ion decreases.

### 3.4. Adsorption balance

#### 3.4.1. Effect of contact time on Fe^2+^ ion removal from wastewater

**Figure 8:**
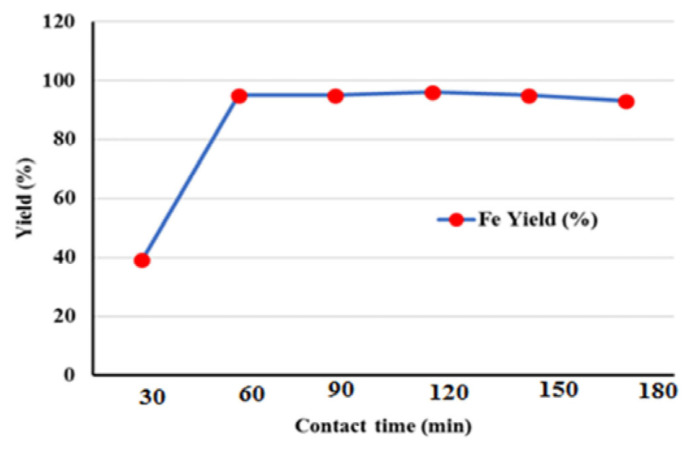
Adsorption kinetics of Fe^2+^ ions depending on the contact time with CBZ-alcohol.

Fe^2+^ ions were tested with CBZ-alcohol at seven time points (0, 30, 60, 90, 120, 150, and 180 min) to determine the adsorption kinetics. The absorption increased rapidly between 30 and 60 min and was stable until 180 min (Figure 8). After the equilibrium phase, the adsorption yield dropped by almost 1%. This suggests that the material field is saturated with Fe^2+^ ions, and then the adsorption stage and the desorption stage is reached. Ho et al. [36] argued that chemical adsorption is more prevalent if it takes less than 3 h to reach the equilibrium, that physical adsorption can be accomplished in 3 to 24 h, and that diffusion processes can be more effective if they take more than 24 h. Overall, it is clear that physical and chemical processes are important in the present study.

#### 3.4.2. Effect of CBZ-alcohol concentration for Fe^2+^ ion removal from wastewater

**Figure 9:**
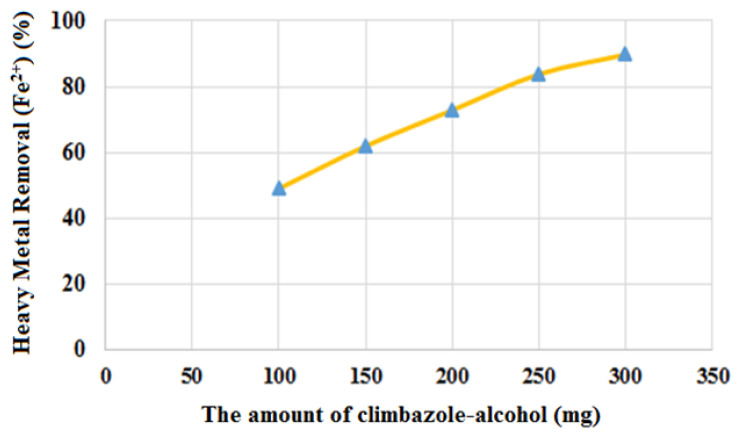
Effect of the amount of CBZ-alcohol on Fe^2+^ ion removal from wastewater.

To find the amount of binding Fe^2+^ ions, wastewater solutions containing 30 ppm Fe^2+^ ions were adjusted with pH 3 buffer. Spectra of the resulting solutions were obtained, and the optimum amount of CBZ-alcohol was determined (Figure 9). When the amount of CBZ-alcohol used was between 100 and 300 mg, Fe^2+^ ion adsorption increased with CBZ-alcohol concentration. Figure 9 shows the adsorption rate proportionally increasing with concentration. In the present study, the effect of temperature on Fe^2+^ ion adsorption was investigated by adjusting the ambient temperature between 40 and 80 °C. At 60 min of contact time and ultrasonic wave velocity of 100 kHz frequency, the best adsorption result was observed at 40 °C. As the temperature increased, Fe^2+^ ion adsorption decreased.

#### 3.4.3. Effect of wastewater volume on Fe^2+^ ion removal from wastewater

**Figure 10:**
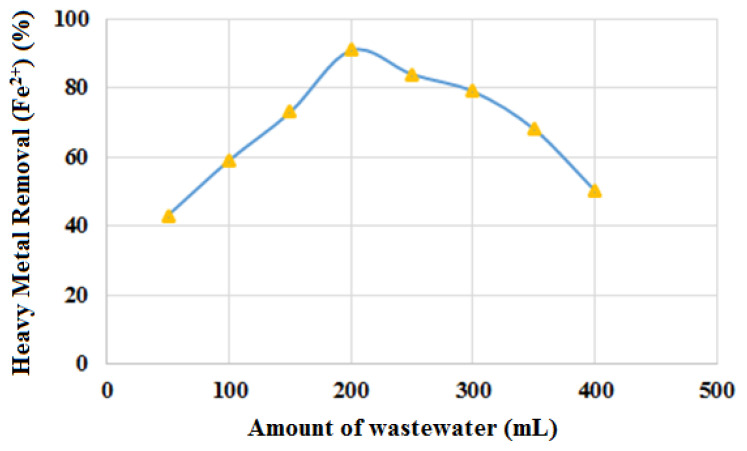
Effect of wastewater volume on Fe^2+^ ion removal from wastewater.

The removal of Fe^2+^ ions from eight different volumes of wastewater (50, 100, 150, 200, 250, 300, 350, and 400 mL) using 300 mg of CBZ-alcohol as adsorbent is shown in Figure 10. The maximum removal was observed in 200 mL of wastewater. When the volume was gradually increased from 200 mL to 400 mL, a decrease in the removal of Fe^2+^ ions was observed (Figure 10). The equilibrium of wastewater after a certain concentration is related to the maximum capacity of the pore structure. An increase in the initial wastewater Fe^2+^ ion concentration may cause an increase in the driving force of the concentration gradient.

#### 3.4.4. Effect of matrix on Fe^2+^ ion removal from wastewater

**Figure 11:**
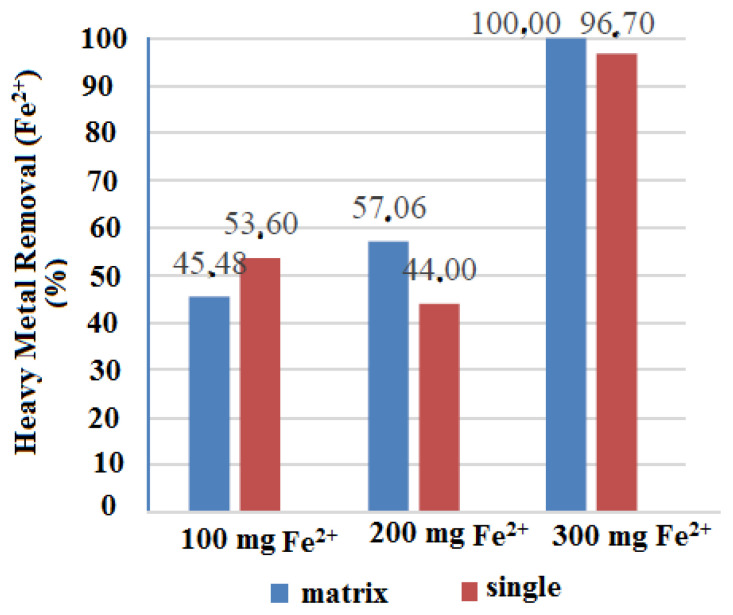
Comparison of Fe^2+^ ion absorption percentage efficiency in matrix and single Fe^2+^ ion solution.

At a lower concentration of Fe^2+^ ions (100 mg), removal was higher in single solution than in matrix (53.6% and 45.48%, respectively). At intermediate (200 mg) and higher (300 mg) concentrations, removal was lower in single solution than in matrix (Figure 11). The diffusion of ions to the magnetite surface may be accelerated at higher concentrations due to chemical potential. Thus, more effective adsorption occurs at higher concentrations in general. In addition, the high absorption in the matrix compared to the solution containing single Fe^2+^ ions demonstrate the ability of CBZ-alcohol to absorb other heavy metal ions.

Adsorption is an attractive alternative to other treatment methods because it is highly efficient and economical for color removal, easily available, complementary to other methods, and requires less biochemical oxygen. In the present study, adsorption was compared with other wastewater treatment methods and the advantages of adsorption are shown in Table 7. Further scale-up research is needed to facilitate the use of these materials in industrial wastewater applications. The reported maximum uptakes of Fe^2+^ ions for the appropriate adsorbent are listed and shown in Table 8.

## 4. Conclusions

In the present study, the efficiency and optimization of CBZ-alcohol as a novel coagulant in the chemical treatment of wastewater with a high Fe^2+^ ion content was investigated. The production of OH–H radicals at the ultrasonic frequency of 100 kHz was carried out by a sonochemical process, but the production of these radicals requires high power and deposition times [42]. The process was optimized using RSM to identify the effects of different process parameters in the chemical pretreatment process. Second-degree models with a high level of confidence (R^2^ > 0.9) can be used to estimate Fe^2+^ ion consumption yields under different conditions. CBZ-alcohol was found to be effective in the pretreatment of wastewater. One of the consequences of climate change today is the reduction in useable water. By applying advanced treatment techniques to wastewater, more water can be recovered, enabling efficient use of water in industry.

The optimum conditions for Fe^2+^ ion removal from wastewater were pH 3, 40 °C, 300 mg of CBZ-alcohol, 200 mL of wastewater, and a contact time of 60 min. The adsorption removal efficiency was 91.83%.

Finally, CBZ-alcohol can be reused for Fe^2+^ ion removal in wastewater treatment. It can adsorb heavy metal in a very short time, and it has been proven to be an efficient adsorbent. Since the present study takes into account the treatment of wastewater, it directly aligns with the United Nations Department of Economic and Social Affairs sustainable development goals 6 (Clean Water and Sanitation), 14 (Life Below Water), and 9 (Industry, Innovation, and Infrastructure).

## Supplementary material

### Spectroscopic data

Figure 12 400 MHz ^1^H-NMR spectra of 1-(4-chlorophenoxy)-1-(1*H*-imidazol-1-yl)-3,3-dimethylbutan-2-olin CDCl_3_.
^1^ H-NMR (400 MHz) spectra of 1-(4-chlorophenoxy)-1-(1*H*-imidazol-1-yl)-3,3-dimethylbutan-2-ol

## Figures and Tables

**Table 1:** Characteristics of wastewater (n = 3).

Parameters	Before adsorption (mean ± SD)	After adsorption (mean ± SD)
pH	8.67	7.05
Conductivity (μS/cm)	2.81	1.28
Nitrogen (mg/L)	97 ± 1	58.43 ± 0.562
Ammonium-nitrogen (mg/L)	93.466 ± 0.318	35.384 ± 1.721
Nitrite-nitrogen (mg/L)	19.621 ± 1.232	3.123
Phosphorus (mg/L)	7.353 ± 0.342	1.896 ± 0.135
Magnesium (mg/L)	105.6 ± 2.674	74.632 ±2.109
Iron (mg/L)	521.02 ± 1.849	1.84 ± 0.027
Chlorine (mg/L)	2.951 ± 0.302	0.747 ± 0.572
Calcium (mg/L)	4.602 ± 0.663	1.682 ± 0.451

**Table 2:** Independent variables and levels used in the Fe^2+^ ion output of wastewater.

Variables	Unit	Code	Levels		
			−1	0	+1
Starting pH		X_1_	1	3	5
Time	min	X_2_	30	45	60
Temperature	°C	X_3_	40	60	80

**Table 3:** Experimental design and Fe^2+^ ion removal experiment results.

Experiment number	Starting pH	Time (min)	Temperature (°C)	Heavy metal removal (Fe^2+^) (%)
1	1	60	80	91.19
2	5	30	80	68.28
3	5	30	40	59.83
4	3	30	60	90.96
5	3	45	60	87.74
6	3	45	40	63.25
7	5	45	60	84.32
8	3	45	60	92.56
9	3	60	60	93.18
10	5	60	80	86.47
11	3	45	80	72.37
12	1	30	80	89.31
13	3	45	60	90.12
14	3	45	60	87.28
15	5	60	40	70.41
16	3	45	60	88.07
17	1	30	40	88.17

**Table 4:** Model R^2^ and R^2^ adj values.

Std. dev.	2.20	R^2^	0.9824
**Mean**	82.56	**Adjusted R** **^2^**	0.9598
**C.V. %**	2.67	**Predicted R** **^2^**	0.6356
		**Adeq precision**	20.9097

**Table 5:** ANOVA results from the quadratic model for percent heavy metal Fe^2+^ ion removal. X_1_: starting pH, X_2_: time (min), X_3_: temperature (°C). Significant at p < 0.05.

Source	Sum of squares	df	Mean square	F-value	p-value	
**Model**	1900.78	9	211.20	43.49	<0.0001	significant
X_1_	64.52	1	64.52	13.29	0.0082	significant
X_2_	76.83	1	76.83	15.82	0.0053	significant
X_3_	106.86	1	106.86	22.01	0.0022	significant
X_1_X_2_	90.39	1	90.39	18.61	0.0035	significant
X_1_X_3_	10.56	1	10.56	2.17	0.1839	not significant
X_2_X_3_	23.13	1	23.13	4.76	0.0654	not significant
X_1_^2^	51.35	1	51.35	10.57	0.0140	significant
X_2_^2^	46.54	1	46.54	9.58	0.0174	significant
X_3_^2^	899.12	1	899.12	185.16	<0.0001	significant
**Residual**	33.99	7	4.86			
Lack of fit	14.77	3	4.92	1.02	0.4706	not significant
Pure error	19.22	4	4.81			
**Cor Total**	1934.77	16				

**Table 6:** Optimum values of parameters used in Fe^2+^ ion adsorption.

Factor	Name	Level	Low level	High level
X_1_	pH	2.95	1.0000	5.00
X_2_	Time	73.91 min	30.00	60.00
X_3_	Temperature	72.95 °C	40.00	80.00

**Table 7:** Comparison of adsorption with other methods in the literature.

Methods of wastewater treatment	Wastewater treatment efficiency (%)
Biological methods	They resist fragmentation. Biological processes are therefore not sufficiently effective for the removal of precipitation metals [37].
Electrochemical methods	The number of experiments on heavy metal removal in wastewater is small. It is also less popular due to the cost of the electricity used [38].
Adsorption	Low-cost adsorbents generally have low adsorption capacities and require large quantities of adsorbent. Therefore, it is the most popular method because it is new, economical, easily available, and provides high efficiency in treatment [39].
Coagulation/ flocculation	Due to its high cost, its applications in wastewater treatment are limited [40].
Chemical precipitation	The generation of large quantities of sludge, high chemical use, and the costs of chemicals limit the use of this process on an industrial scale [41].

**Table 8:** Fe^2+^ ion adsorption on nanoporous adsorbents with the highest capacity.

Adsorbent	Adsorption capacity (%)
Graphene oxide-ordered mesoporous silica materials	78.7 [10]
Nanomaterials	88.6 [12]
Carbon nanostructured materials	90.1 [13]
Nutshell as biosorbent	86.5 [24]
Nanocomposite	84.3 [39]
**CBZ-alcohol**	**91.83** (The present study)
