# Supplementary figures and images for: Removal of heavy metal iron(II) ions from wastewater using an ultrasonic system with climbazole-alcohol
Source: Turk J Chem. 2025 Mar 12;49(3):279–92. doi: 10.55730/1300-0527.3729 (PMC12253972; doi:10.55730/1300-0527.3729)

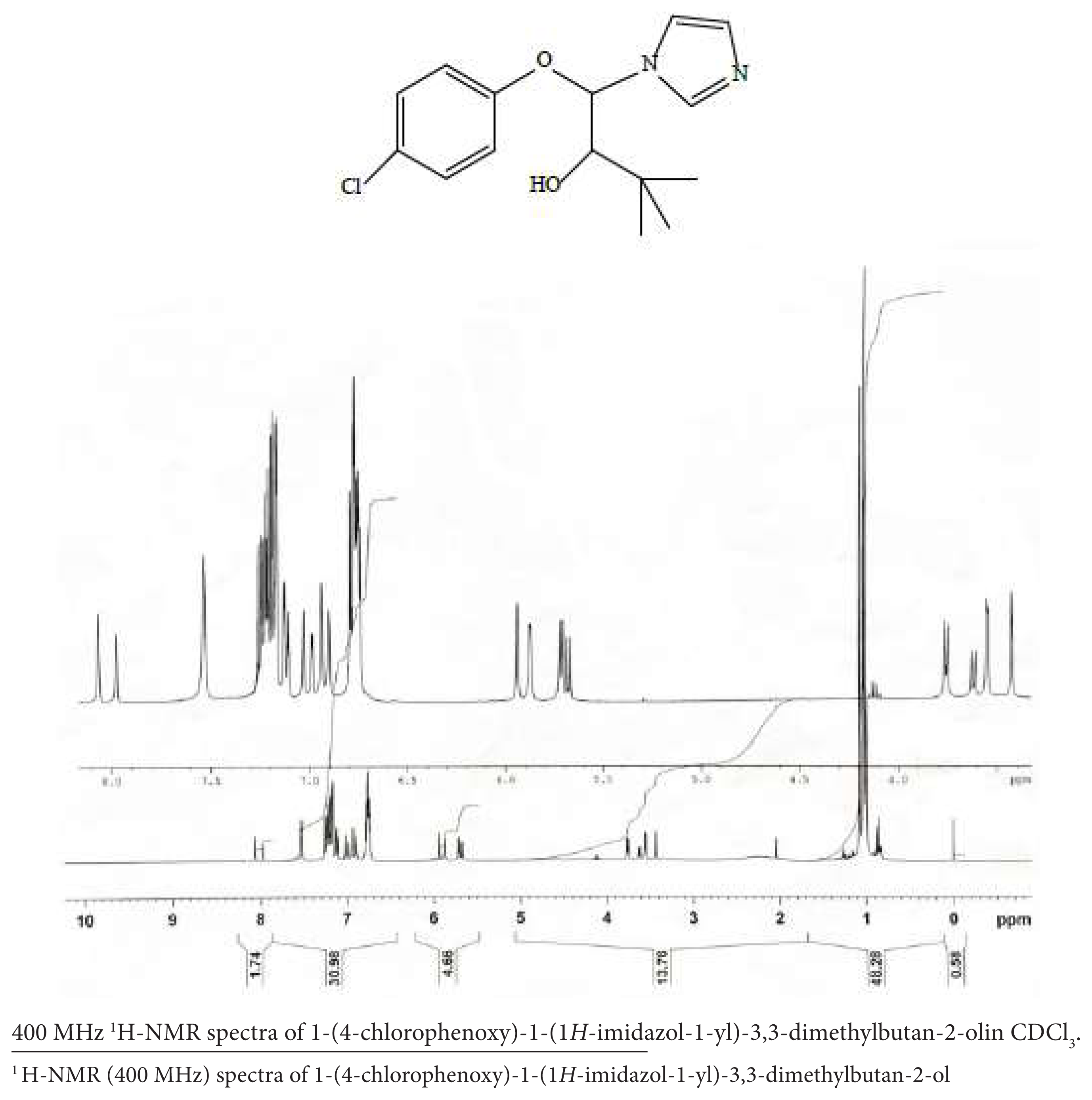

Supplement: Figure 12 — 400 MHz 1H-NMR spectra of 1-(4-chlorophenoxy)-1-(1H-imidazol-1-yl)-3,3-dimethylbutan-2-olin CDCl3. 1 H-NMR (400 MHz) spectra of 1-(4-chlorophenoxy)-1-(1H-imidazol-1-yl)-3,3-dimethylbutan-2-ol [file tjc-49-03-279s1.tif]
